# Kidney Stones and Risk of Osteoporotic Fracture in Chronic Kidney Disease

**DOI:** 10.1038/s41598-018-38191-1

**Published:** 2019-02-13

**Authors:** Seung Gyu Han, Jieun Oh, Hee Jung Jeon, Chan Park, Jeonghwan Cho, Dong Ho Shin

**Affiliations:** 1 Department of Internal Medicine, Kang Dong Dr. Han medical clinic, 156, Seongan-ro, Gangdong-gu, Seoul 05355 Korea; 2 Department of Internal Medicine, College of Medicine, Hallym University, Kandong Sacred Heart Hospital, 150, Seongan-ro, Gangdong-gu, Seoul 05355 Korea

## Abstract

Osteoporotic fracture associated with calcium dysregulation is more common in patients with kidney stones. However, little is known about the association of kidney stones and bone health status in patients with chronic kidney disease (CKD). This retrospective medical record-based study included 2282 patients with stable stage 3–4 CKD between 2007 and 2017. Of these, 113 patients were diagnosed with kidney stones. Propensity score matching for 226 patients with and without kidney stones showed that osteoporotic fracture occurred more often in patients with kidney stones (33, 29.2%) than in patients without kidney stones (16, 14.2%), resulting in rates of 5.56 and 2.63/100 patient-years, respectively (p < 0.01). In particular, Cox proportional hazard analysis revealed that kidney stones were significantly associated with osteoporotic fracture, even after adjusting for age, sex, body mass index, kidney stones, estimated glomerular filtration rate, excessive alcohol consumption, current smoking, and steroid use in patients with CKD stage 3–4 (hazard ratio, 2.32, 95% CI 1.24–4.34, p = 0.01). This study showed that the presence of kidney stones was a significant predictor for osteoporotic fracture in patients with CKD, suggesting that it should be considered as a clinical risk factor for osteoporotic fracture in them.

## Introduction

Osteoporotic fracture in patients with chronic kidney disease (CKD) results in high morbidity and mortality^1^, and this imposes a high economic burden on society. Generally, CKD patients have a higher osteoporotic fracture risk compared with the general population^2^. Bone changes in patients with CKD are characterized by abnormalities of bone turnover, mineralization, volume, linear growth, and strength^3^. Therefore, it is important to predict osteoporotic fracture risk in these patients. Although revised Kidney Disease Improving Global Outcomes guidelines recommend bone mineral density (BMD) measurement with dual-energy X-ray absorptiometry (DXA) to assess osteoporotic fracture risk in these patients^4^, the ability to predict fracture risk remains limited. Bone strength is determined by both the density and quality of bone. However, DXA BMD only measures the density of bone by assessing the amount of bone mineral. To overcome this limitation, high-resolution peripheral quantitative computed tomography and bone turnover markers such as osteocalcin, bone-specific alkaline phosphatase, procollagen type-1 N-terminal propeptide, and tartrate-resistant acid phosphatase-5b have been used to help predict osteoporotic fracture risk^5,6^. However, their application in clinical practice is limited in terms of medical cost and reimbursement. Therefore, because the aetiology of bone changes is complex in patients with CKD^7^, clinical risk factors should be considered, rather than relying solely on fragmented test data to predict osteoporotic fracture. Generally, factors other than BMD that affect osteoporotic fracture risk include advancing age, previous fracture, glucocorticoid therapy, family history of hip fracture, low body weight, excessive alcohol consumption, and current smoking^8–11^.

Most kidney stones result from hyercalciuria, which is caused by systemic dysregulation of calcium homeostasis^12^. Osteoporotic fractures are also associated with systemic dysregulation of calcium homeostasis, including intestinal calcium absorption, renal tubular calcium reabsorption, and bone demineralization^13^. Therefore, if patients with hypercalciuria excrete more calcium than they absorb, reduced bone density can occur. In fact, some previous studies reported that individuals with kidney stones were at higher risk for osteoporotic fracture^14–17^.

Conceptually, CKD can prevent kidney stone formation because of significant reduction in urine calcium excretion, an important risk factor for stone formation^18^. Interestingly, there is evidence that that the recurrence rate of kidney stones is lower in stone formers with reduced kidney function^19^. Therefore, the occurrence of kidney stones in patients with CKD may indicate severe dysregulation of calcium homeostasis. However, little is known about the association of kidney stones and bone health status in these patients. Therefore, it was hypothesized that the occurrence of kidney stones would be related with an increased risk of poor bone health in patients with CKD. In addition, we investigated whether the occurrence of kidney stones in these patients was associated with higher risk of osteoporotic fracture.

## Results

### Kidney stone characteristics

This study included 113 patients with stage 3–4 CKD and kidney stones. Of these, 56, 34, and 14 had nephrolithiasis, ureterolithiasis, or cystolithiasis, respectively. The rest were diagnosed with passed kidney stones. At study enrolment, 94 patients were diagnosed with asymptomatic kidney stones. In addition, 19 patients were diagnosed with symptomatic kidney stones. Of note, the median time interval between study enrolment and the diagnosis of symptomatic kidney stones was 9 months (interquartile range, 3–18 months).

### Patient characteristics

Baseline characteristics of patients with and without kidney stones and characteristics after propensity score matching are shown in Table 1. A total 2282 patients with stage 3–4 CKD were included in the study. Compared to those without kidney stones, more patients with stones were men (73.5% vs. 59.0%, *p* = 0.003), with higher body mass index (BMI) (21.0 ± 0.8 vs. 20.9 ± 0.7, *p* = 0.04) and estimated glomerular filtration rate (eGFR) (37.3 ± 19.3 vs. 31.6 ± 22.2 ml/min/1.73 m^2^, *p* = 0.003). In addition, use of vitamin D supplements (36.3% vs. 26.5%, *p* = 0.03) and calcium supplements (34.5% vs. 25.7%, *p* = 0.05) was more common in patients with kidney stones. However, serum phosphate concentration was lower in those with kidney stones. On the other hand, the proportion of osteoporotic fracture was higher in patients with kidney stones than in patients without kidney stones (33, 29.2% vs. 376, 17.3%, *p* = 0.01). Of note, there were 409 occurrences of osteoporotic fracture during follow-up, including 72 hip, 77 forearm, 29 humeral, 201 vertebral, and 30 other fractures. Especially, compared to patients without kidney stones, the relative frequency of hip and vertebral fractures was higher in patients with kidney stones (Supplement Table S1).

**Table 1:** Characteristics of the study population before and after propensity score matching.

	Before propensity score matching			After propensity score matching		
	Without kidney stones (n = 2169)	With kidney stones (n = 113)	*p*-value	Without kidney stones (n = 113)	With kidney stones (n = 113)	*p*-value
Korean (%)	2169 (100)	113 (100)	1	113 (100)	113 (100)	1
Male (%)	1279 (59.0)	83 (73.5)	0.003	89 (78.8)	83 (73.5)	0.44
Age (years)	64.8 ± 14.2	65.6 ± 11.9	0.52	65.1 ± 14.0	65.6 ± 11.9	0.78
Weight (kg)	56.7 ± 8.2	58.4 ± 7.7	0.03	59 ± 7.6	58.4 ± 7.7	0.62
Height (m)	1.6 ± 0.4	1.7 ± 0.5	0.02	1.7 ± 0.1	1.7 ± 0.5	0.54
BMI	20.9 ± 0.7	21.0 ± 0.8	0.04	21.0 ± 0.6	21.0 ± 0.7	0.91
**Cause of CKD**						
Diabetes (%)	881 (40.6)	56 (49.6)	0.07	55 (48.7)	56 (49.6)	0.99
Non-diabetes (%)	1288 (59.4)	57 (50.4)		58 (51.3)	57 (50.4)	
**Laboratory parameters**						
eGFR (ml/min/1.73 m^2^)	31.6 ± 22.2	37.3 ± 19.3	0.003	41.0 ± 20.1	38.1 ± 17.2	0.25
CKD stage 3 (%)	1048 (48.3)	73 (64.6)	0.001	74 (65.5)	73 (64.6)	0.89
CKD stage 4 (%)	1121 (51.7)	40 (35.4)		39 (34.5)	40 (35.4)	
serum calcium (mg/dl)	9.1 ± 0.8	9.0 ± 0.7	0.09	9.1 ± 0.7	9.0 ± 0.7	0.46
serum phosphate (mg/dl)	3.9 ± 0.9	3.7 ± 0.6	0.05	3.9 ± 0.9	3.7 ± 0.9	0.26
serum bicarbonate (mmol/l)	24.9 ± 2.0	24.5 ± 1.9	0.05	24.9 ± 2.0	24.5 ± 2.1	0.11
serum PTH (pg/ml)	95.9 ± 18.4	96.4 ± 18.9	0.80	95.7 ± 18.0	96.4 ± 18.9	0.77
Current alcohol consumption (%)	232 (10.7)	13 (11.5)	0.91	8 (7.1)	13 (11.5)	0.36
Current smoking (%)	147 (6.8)	6 (5.3)	0.68	11 (9.7)	6 (5.3)	0.31
Use of vitamin D supplement (%)	575 (26.5)	41 (36.3)	0.03	31 (27.4)	41 (36.3)	0.20
Use of calcium supplement (%)	557 (25.7)	39 (34.5)	0.05	31 (27.4)	39 (34.5)	0.31
Use of phosphate binder (%)	253 (11.7)	12 (10.6)	0.85	17 (15.0)	12 (10.6)	0.43
Use of steroid (%)	34 (1.6)	2 (1.8)	0.87	3 (2.7)	2 (1.8)	0.99

Values are expressed as mean ± SD or number (percentage).

BMI: body mass index, CKD: chronic kidney disease, CKD stage 3: 30–59 ml/min/1.73 m^2^, CKD stage 4: 15–29 ml/min/1.73 m^2^, eGFR: estimated glomerular filtration rate, PTH: parathyroid hormone.

After propensity score matching, the 2 groups had similar characteristics. Of the 226 patients in the matched analysis, 172 (76.1%) were men and the average age was 65.3 years. In addition, the mean eGFR was 38.9 ± 15.4 ml/min/1.73 m^2^.

### Osteoporotic fracture characteristics after propensity score matching

Of 49 osteoporotic fractures, 5 involved the hip, 11 the forearm, and 33 the vertebrae. In patients with kidney stones, osteoporotic fractures occurred in 24 and 9 patients after a diagnosis of asymptomatic or symptomatic kidney stones, respectively. Of note, there was no association between fracture site and symptomatic vs. asymptomatic kidney stones (Supplement Table S2).

### Incidence of osteoporotic fracture

**Figure 1:**
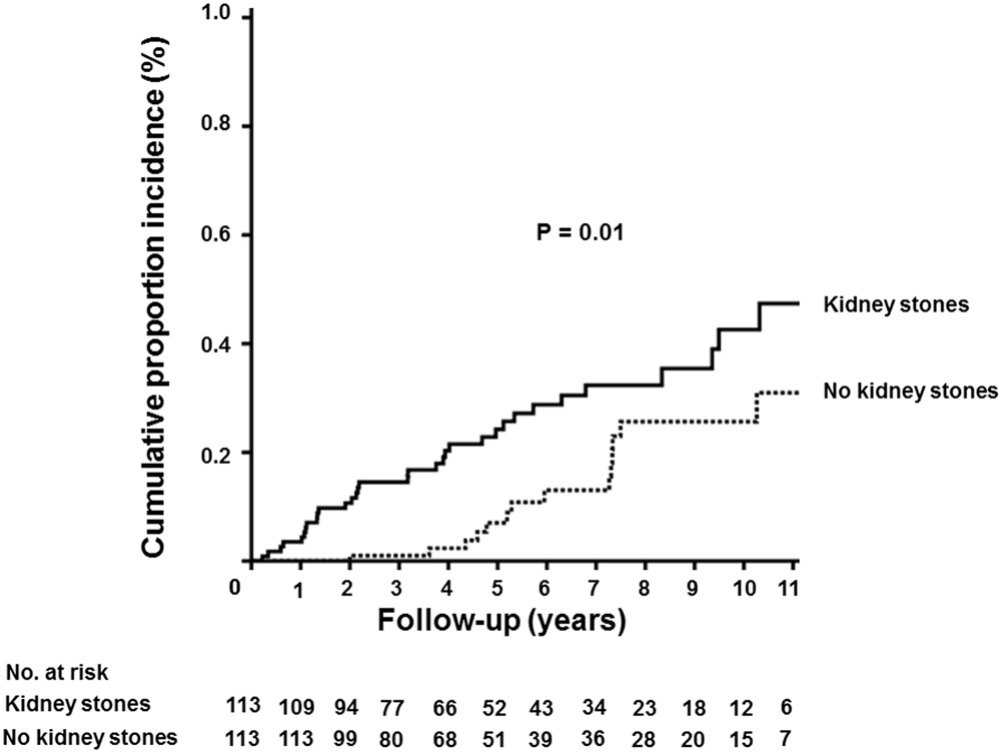
Cumulative proportion with osteoporotic fracture according to presence of kidney stones. Patients with kidney stones had a significantly higher incidence of osteoporotic fracture (*p* = 0.01).

During a mean follow-up duration of 64.7 ± 38.7 months, osteoporotic fracture occurred more often in patients with kidney stones (33, 29.2%) than in patients without kidney stones (16, 14.2%), resulting in rates of 5.56 and 2.62/100 patient-years, respectively (*p* < 0.01) (Table 2). The odds ratio for osteoporotic fracture in patients with kidney stones was 2.50 [98% confidence interval (CI), 1.28–4.87; *p* = 0.01]. Kaplan-Meier analysis also indicated that osteoporotic fractures were significantly more common in patients with kidney stones (Fig. 1, *p* = 0.01).

**Table 2:** Incidence of osteoporotic fracture according to presence of kidney stones.

	Without kidney stones		With kidney stones		Odds ratio (95% CI)	*p*-value
	n (%)	Rate (100 patient-years)	n (%)	Rate (100 patient-years)		
Osteoporotic fracture	16 (14.2)	2.62	33 (29.2)	5.56	2.50 (1.28–4.87)	0.006

### Predictive value of kidney stones for osteoporotic fracture

Risk factors for osteoporotic fracture are analysed in Table 3. Variables that were related to osteoporotic fracture in univariate Cox regression analysis were male sex, age, kidney stones, eGFR, and steroid use. Kidney stones were significantly associated with osteoporotic fracture, even after adjustment for age, sex, BMI, kidney stones, eGFR, excessive alcohol consumption, current smoking, and steroid use (hazard ratio [HR], 2.32; 95% CI, 1.24–4.34; *p* = 0.01).

**Table 3:** Hazard ratio for osteoporotic fracture after propensity score matching.

Variables	Univariate	*p*-value	Multivariate	*p*-value
	Hazard ratio (95% CI)		Hazard ratio (95% CI)	
Male (vs. female)	0.51 (0.28–0.92)	0.03	0.70 (0.38–1.30)	0.26
Age (years)	1.03 (1.00–1.06)	0.02	1.04 (1.01–1.07)	0.002
BMI	0.75 (0.48–1.17)	0.20	0.71 (0.44–1.13)	0.14
Kidney stones	2.13 (1.17–3.87)	0.01	2.32 (1.24–4.34)	0.01
Diabetes	1.06 (0.60–1.86)	0.84		
eGFR (ml/min/1.73 m^2^)	0.98 (0.97–1.00)	0.05	0.98 (0.96–0.99)	0.02
serum calcium	0.85 (0.58–1.33)	0.87		
serum phosphate	0.85 (0.62–1.17)	0.32		
serum bicarbonate	0.99 (0.86–1.14)	0.92		
serum PTH	0.99 (0.98–1.01)	0.34		
Current alcohol consumption	1.01 (0.40–2.55)	0.99	0.88 (0.34–2.29)	0.80
Current smoking	0.83 (0.26–2.69)	0.76	0.97 (0.29–3.22)	0.96
Use of vitamin D supplement	1.51 (0.86–2.66)	0.16		
Use of calcium supplement	1.30 (0.73–2.30)	0.38		
Use of calcium phosphate binder	1.01 (0.040–2.55)	0.99		
Use of steroid	12.7 (2.94–55.11)	0.001	7.64 (1.70–34.40)	0.01

BMI: body mass index, CKD: chronic kidney disease, eGFR: estimated glomerular filtration rate, PTH: parathyroid hormone.

### Synergic effect on osteoporotic fracture between kidney stones and renal function

Compared to patients without kidney stones and with CKD stage 3, the multi-adjusted HR of osteoporotic fracture was highest in patients with kidney stones and CKD stage 4. In addition, the RERI and AP scales were positive, and the synergy index was >1, meaning a positive additive interaction effect on osteoporotic fracture between kidney stones and renal function (Table 4).

**Table 4:** Interaction analysis between kidney stones and renal function in osteoporotic fracture.

Kidney stones	Renal function	Total number	Cases of osteoporotic fracture	Hazard ratio (95% CI)^a^	*p-*value
No	CKD stage 3	74	4	1	
Yes	CKD stage 3	73	12	3.13 (1.01–9.81)	0.05
No	CKD stage 4	39	12	4.78 (1.51–15.27)	0.008
Yes	CKD stage 4	40	21	11.42 (3.78–34.47)	<0.001

Measurement of interaction on additive scale: RERI (95% CI) = 3.78 (1.95–5.61); AP = 0.47 (0.34–0.61); SI = 2.18 (1.33–3.56).

RERI: relative excess risk due to interaction, AP: attributable proportion due to interaction, SI: synergy index.

^a^Adjusted for sex, age, BMI, current alcohol consumption, current smoking, and use of steroid.

CKD stage 3: 30–59 ml/min/1.73 m^2^, CKD stage 4: 15–29 ml/min/1.73 m^2^.

## Discussion

Despite previous reports that kidney stones were associated with higher risk of osteoporotic fracture, the association of kidney stones and osteoporotic fracture has not been established in patients with CKD. The result of this study showed that kidney stones were significantly associated with higher risk of osteoporotic fracture in patients with CKD after adjusting for most conventional fracture predictors except BMD. Our findings suggest that the presence of kidney stones should be considered as a clinical risk factor for osteoporotic fracture in patients with CKD.

During follow-up, there were 409 incidents of osteoporotic fracture in the unmatched CKD cohort (10.7/100 person-years). In line with the findings that fracture rates are elevated in patients with CKD, this rate is consistent with that reported by other studies^16,20^. Interestingly, in this cohort, the incidence rate of osteoporotic fracture was higher in patients with kidney stones than in patients without kidney stones. However, because the heterogeneity of baseline characteristics could cause confusion in determining the association of kidney stones and osteoporotic fracture, propensity score matching was used in this cohort to minimize selection bias. After propensity score matching, kidney stones were a significant risk factor for osteoporotic fracture in a multivariate model.

Although the prevalence of kidney stones has varied by region or time of survey, 8.8% was reported in the National Health and Nutritional Examination Survey for 2007 to 2010^21^. Worldwide, about 80% of kidney stones are composed of calcium in the form of either calcium oxalate or calcium phosphate^22^. These stones develop when urine becomes supersaturated with insoluble compounds containing calcium, resulting from hypercalciuria^23^. The kidney plays a critical role in regulating serum calcium levels^24^. Calcium is both filtered and reabsorbed in the kidney^24^. If filtered calcium is abnormally increased or reabsorbed, the serum level is reduced and hypercalciuria occurs^25^. Therefore, reduced serum calcium levels stimulate increased calcium flow into plasma from bone to maintain equilibrium^26^. Eventually, hypercalciuria is associated with increased bone resorption, which leads to negative calcium balance and the loss of bone. Taken together, the presence of kidney stones, which are related to hypercalciuria, is considered to be the most common identifiable metabolic risk factor for osteopenia and osteoporosis. In fact, several studies showed that osteoporotic fracture occurred more frequently in patients with kidney stones than in the general papulation^14–17^. Meanwhile, the effect of CKD on the incidence of kidney stones has not been well studied. Conceptually, decreased filtered calcium caused by reduced GFR would lead to decreased supersaturation of calcium salts. In a longitudinal study of 3266 patients with kidney stones, Worcester *et al*. reported a decreased risk of recurrent kidney stones in patients with a single kidney compared to those with both kidneys intact^19^. In line with this finding, the prevalence of kidney stones in the present study was 4.5%, which was lower than in a previous report^21^.

The development of kidney stones in CKD despite preventive measures indicates that tubular calcium reabsorption is reduced. Approximately 60–70% of filtered calcium reabsorption occurs in the proximal convoluted tubule, with 20% in the loop of Henle, 10% in the distal convoluted tubule, and 5% in the collecting duct^24^. In contrast to the passive calcium reabsorption that occurs in the more proximal nephron, active calcium reabsorption in the distal nephron is stimulated by parathyroid hormone (PTH)^24^. As kidney function decreases below a certain threshold value, PTH levels gradually increase^27,28^. Therefore, the development of kidney stones despite elevated PTH levels in patients with CKD may reflect a problem with PTH or calcium sensing receptor function in the renal tubule. Interestingly, Pubali *et al*. recently showed an association between PTH gene polymorphisms and increased risk of kidney stones^29^. Because PTH affects bone microarchitecture or turnover, most studies on PTH gene polymorphisms have focused on osteoporosis, bone mass, and bone size.

The Fracture Risk Assessment Tool (FRAX) is used to predict bone fracture^30^. FRAX integrates clinical risk factor such as advancing age, previous fracture, glucocorticoid therapy, parental history of hip fracture, low body weight, current cigarette smoking, excessive alcohol consumption, rheumatoid arthritis, secondary osteoporosis, and BMD at the femoral neck^31^. However, this model does not include any adjustment of risk according to eGFR. In addition, although DXA is the most commonly used technique to assess BMD in patients with and without CKD, it cannot assess bone microarchitecture or bone turnover. Therefore, the presence of kidney stones can affect bone physiology and may be a clinical predictor of osteoporotic fracture in CKD patients.

There are several limitations to our study. First, even though propensity score matching was used, this was a retrospective single-centre study based on a small number of CKD patients with kidney stones. Second, some patients who passed kidney stones might not have been diagnosed. Third, analysis of kidney stone composition was not performed. Fourth, parental history of hip fracture and previous fracture were not investigated. In addition, there was no measurement of BMD with DXA. Lastly, PTH gene polymorphisms or calcium sensing receptor function in the renal tubule were not also studied.

In conclusion, there is a significant association between kidney stones in CKD patients and osteoporotic fracture after adjusting for most conventional fracture predictors except BMD. Accordingly, the presence of kidney stones should be considered a factor that contributes to osteoporotic fracture risk. A large-scale prospective trial is needed to confirm this conclusion.

## Methods

### Ethics statement

This study was performed in accordance with the Declaration of Helsinki and approved by the Institutional Review Board (IRB) of Kangdong Sacred Heart Hospital, (Refs. Kangdong 2018-07-014). This was a retrospective medical record-based study and the study subjects were de-identified. The IRB waived the need for written consent from the patients.

### Patients

**Figure 2:**
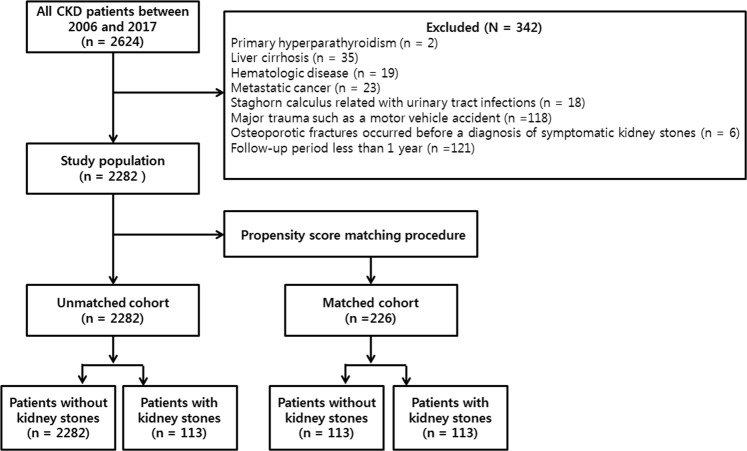
Patient selection and study flow.

For this study, we initially selected 2624 patients with clinically stable stage 3–4 CKD at Kangdong Sacred Heart hospital between January 2006 and December 2017. Patients with the following conditions were excluded: Primary hyperparathyroidism (n = 2), liver cirrhosis (n = 35), haematologic disease (n = 19), metastatic cancer (n = 23), staghorn calculus associated with urinary tract infection (n = 18), fracture caused by major trauma such as a motor vehicle accident (n = 118), osteoporotic fracture before a diagnosis of symptomatic kidney stones (n = 6), and a follow-up period less than 1 year (n = 121). The remaining 2282 patients were included in this study (Fig. 2).

### Data collection

When stable stage 3–4 CKD patients first visited the nephrology clinic or renal function of existing follow-up patients with early stage CKD reached stage 3–4, baseline characteristics, including demographic, laboratory, and clinical data were collected. Stable stage 3–4 CKD patients who first visited the nephrology clinic underwent abdominal ultrasound, X-ray, or non-contrast computed tomography (CT). In addition, when renal function reached stage 3–4 in existing follow-up patients with early stage CKD, the above-mentioned imaging studies were repeated.

This study included patients with symptomatic and asymptomatic kidney stones. Kidney stones were of 3 types, according to location: nephrolithiasis (in the kidney), ureterolithiasis (in the ureter), cystolithiasis (in the bladder). The diagnosis was confirmed with imaging studies. In addition, when kidney stones were not diagnosed on baseline imaging, additional studies were performed for symptoms of renal colic accompanied by urinary urgency, restlessness, haematuria, sweating, nausea, and vomiting during the follow-up period. Exceptionally, patients were diagnosed with kidney stones without radiologic confirmation, based on a clinical presentation consistent with having passed a stone.

The study outcome was the occurrence of osteoporotic fracture, defined as fracture occurring with a fall from a standing height or less, without major trauma such as a motor vehicle accident. Osteoporotic fracture occurrence was defined as the first surgical or nonsurgical fracture requiring hospitalization or outpatient treatment during the study follow-up period. Of note, plain radiographs or magnetic resonance imaging (MRI) were available for each case of fracture and the diagnosis of fracture was confirmed based on the radiologist’s report. Excessive alcohol consumption was defined as drinking 3 or more units of alcohol daily. Current tobacco use was reported as smoking tobacco at the time of study enrolment. Vitamin D supplementation was defined as any use of alfacalcidol, cholecalciferol, or calcitriol. Phosphate binder included calcium acetate or calcium carbonate. Corticosteroid use was defined as a dose of 5 mg daily or more (or equivalent dose of other glucocorticoid). Of note, patients who received a vitamin D supplement, calcium supplement, phosphate binder, or corticosteroids were defined as those treated for >3 months.

### Propensity score matching

The propensity score was determined using a multivariable logistic regression model with baseline covariates (Table 1). Propensity score matching was performed using the MatchIt package in R. Patients with the nearest propensity scores in 2 groups (patients with or without kidney stones) were matched using a 1:1 scheme without replacement using a greedy algorithm. Propensity score matching allowed comparison of outcomes between individuals with a similar likelihood.

### Statistical analyses

Statistical analyses were performed using SPSS 19.0 (SPSS Inc., Chicago, IL, USA). The Kolmogorov-Smirnov test was used to test the normality of continuous variables. The normally distributed variables were expressed as mean ± SD and compared with Student’s *t*-test for 2 groups. The categorical variables were expressed as frequencies and percentages and compared with either the chi-square test or Fisher’s exact test. Cumulative survival curves were generated using the Kaplan-Meier method to determine the association of kidney stones and osteoporotic fracture, and between-group osteoporotic fracture was compared using a log-rank test. The prognostic value of kidney stones for osteoporotic fracture was determined using multivariate Cox proportional-hazards regression analysis, which included all covariates with *p* values < 0.05 in the univariate analysis or conventional fracture risk factors other than BMD. To evaluate a potential interaction between kidney stones and renal function in osteoporotic fracture, patients were divided into 4 subgroups by kidney stones and renal function (CKD stage 3 vs. CKD stage 4). The relative excess risk due to interaction (RERI), the attributable proportion due to interaction (AP), and the synergic index (SI) were used to test an interaction on an additive scale^32^. Both the point estimation and 95% CI of RERI, AP, and SI were assessed using a method accounting for asymmetric distribution of confidence limits for risk ratio^33^.

## Supplementary information

Kidney Stones and Risk of Osteoporotic Fracture in Chronic Kidney Disease

## Data Availability

The datasets generated during and/or analysed during the current study are available from the corresponding author on reasonable request.
