# Supplementary material for: Kidney Stones and Risk of Osteoporotic Fracture in Chronic Kidney Disease
Source: Sci Rep. 2019 Feb 13;9:1929. doi: 10.1038/s41598-018-38191-1 (PMC6374417; doi:10.1038/s41598-018-38191-1)
Supplement: Supplementary file 1 — Kidney Stones and Risk of Osteoporotic Fracture in Chronic Kidney Disease [file 41598_2018_38191_MOESM1_ESM.docx]

**Kidney Stones and Risk of Osteoporotic Fracture in Chronic Kidney Disease**

Seung Gyu Han, M.D. ^1^, Jieun Oh, M.D., Ph.D. ^2^, Hee Jung Jeon, M.D. ^2^, Chan Park, M.D. ^2^, Jeonghwan Cho, M.D. ^2^, Dong Ho Shin, M.D., Ph.D. ^2,*^

^1^Department of Internal Medicine, Kang Dong Dr. Han medical clinic, 156, Seongan-ro, Gangdong-gu, Seoul 05355 Korea

^2^Department of Internal Medicine, College of Medicine, Hallym University, Kandong Sacred Heart Hospital, 150, Seongan-ro, Gangdong-gu, Seoul 05355 Korea

**Supplementary Information**

**Table S1.** The relative frequency of fracture sites in patients with and without kidney stones

**Table S2.** The relative frequency of fracture sites in patients with asymptomatic and symptomatic kidney stones

Table S1. The relative frequency of fracture sites in patients with and without kidney stones

|  | Total | Without kidney stones | With kidney stones | p-value |
| --- | --- | --- | --- | --- |
|  | (n = 2282) | (n = 2169) | (n = 113) |  |
| Fracture site |  |  |  |  |
| Humerus (%) | 29 (1.3) | 26 (1.2) | 3 (2.7) | 0.17 |
| Forearm (%) | 77 (3.4) | 72 (3.3) | 5 (4.4) | 0.24 |
| Vertebrae (%) | 201 (8.8) | 184 (8.5) | 17(15.0) | 0.016 |
| Hip (%) | 72 (3.2) | 64 (3.0) | 8 (7.1) | 0.014 |
| Others (%) | 30 (1.3) | 30 (1.4) | 0 (0) | 0.4 |

Table S2. The relative frequency of fracture sites in patients with asymptomatic and symptomatic kidney stones

|  | Total | Asymptomatic kidney stones | Symptomatic kidney stones | p-value |
| --- | --- | --- | --- | --- |
|  | (n = 113) | (n = 94) | (n = 19) |  |
| Fracture site |  |  |  |  |
| Forearm (%) | 3 (2.7) | 2 (2.1) | 1 (5.3) | 0.43 |
| Vertebrae (%) | 24 (21.2) | 18 (19.1) | 6 (31.6) | 0.23 |
| Hip (%) | 6 (5.3) | 4 (4.3) | 2 (10.5) | 0.27 |
